# Rac1 Guides Porf-2 to Wnt Pathway to Mediate Neural Stem Cell Proliferation

**DOI:** 10.3389/fnmol.2017.00172

**Published:** 2017-06-02

**Authors:** Xi-Tao Yang, Guo-Hui Huang, Hong-Jiang Li, Zhao-Liang Sun, Nan-Jie Xu, Dong-Fu Feng

**Affiliations:** ^1^Department of Neurosurgery, Shanghai Ninth People’s Hospital, Shanghai Jiao Tong University School of MedicineShanghai, China; ^2^Institute of Traumatic Medicine, Shanghai Jiao Tong University School of MedicineShanghai, China; ^3^Department of Interventional Radiotherapy, Shanghai Ninth People’s Hospital, Shanghai Jiao Tong University School of MedicineShanghai, China; ^4^Neuroscience Division, Department of Anatomy, Histology and Embryology, Shanghai Jiao Tong University School of MedicineShanghai, China; ^5^Shanghai Key Laboratory for Tumor Microenvironment and Inflammation, Department of Biochemistry and Molecular Cell Biology, Shanghai Jiao Tong University School of MedicineShanghai, China; ^6^Key Laboratory of Cell Differentiation and Apoptosis of the Chinese Ministry of Education, Shanghai Jiao Tong University School of MedicineShanghai, China

**Keywords:** RhoGAPs, Porf-2, Wnt/β-catenin pathway, neural stem cells, optic nerve injury

## Abstract

The molecular and cellular mechanisms underlying the anti-proliferative effects of preoptic regulator factor 2 (Porf-2) on neural stem cells (NSCs) remain largely unknown. Here, we found that Porf-2 inhibits the activity of ras-related C3 botulinum toxin substrate 1 (Rac1) protein in hippocampus-derived rat NSCs. Reduced Rac1 activity impaired the nuclear translocation of β-catenin, ultimately causing a repression of NSCs proliferation. Porf-2 knockdown enhanced NSCs proliferation but not in the presence of small molecule inhibitors of Rac1 or Wnt. At the same time, the repression of NSCs proliferation caused by Porf-2 overexpression was counteracted by small molecule activators of Rac1 or Wnt. By using a rat optic nerve crush model, we observed that Porf-2 knockdown enhanced the recovery of visual function. In particular, optic nerve injury in rats led to increased Wnt family member 3a (Wnt3a) protein expression, which we found responsible for enhancing Porf-2 knockdown-induced NSCs proliferation. These findings suggest that Porf-2 exerts its inhibitory effect on NSCs proliferation via Rac1-Wnt/β-catenin pathway. Porf-2 may therefore represent and interesting target for optic nerve injury recovery and therapy.

## Introduction

The loss of retinal ganglion cells, caused by various diseases including optic nerve injury, results in visual dysfunction and can even cause blindness (Berkelaar et al., 1994; Quigley et al., 1995; Bien et al., 1999). Unfortunately, the standard therapeutic strategy for the treatment of optic nerve injury is absent. Neural stem cells (NSCs) can be isolated, proliferated, genetically manipulated and differentiated and reintroduced into pathologically altered central nerve system (CNS), which have been considered a potential therapeutic approach that may restore or sustain retinal function and prevent blindness in patients with optic nerve injury (Nishida et al., 2000; Banin et al., 2006; Harvey et al., 2006; Muller et al., 2006; Bi et al., 2009; Yang et al., 2014). However, engrafted NSCs usually could differentiate into glial cells rather than neurons *in vivo*, limiting their therapeutic effects (Zhou et al., 2009). Moreover, poor survival and poor proliferation rate have also limited the practical use of NSCs-based therapy (Lu et al., 2008; Carreira et al., 2014). Therefore, identifying molecules that can inhibit NSCs proliferation and deciphering the underlying cellular and molecular mechanisms of NSCs proliferation may help in the development of NSCs-based therapies.

Preoptic regulatory factor-2 (Porf-2), also named as Cross GTPase-activating protein (CrossGAP)/Vilse, was first discovered from the preoptic area of the hypothalamus of the castrated male rat (Nowak, 1990). Porf-2 can strongly and specifically down-regulate the activity of Rac1 to mediate midline axon guidance and dendritic spine formation (Lundstrom et al., 2004; Hu et al., 2005; Lim et al., 2014). Previously, we have demonstrated the role of Porf-2 in NSCs proliferation possibly via Wnt signaling (Huang et al., 2016). However, whether and how Porf-2 functions through Wnt are still not fully determined. The molecular interaction or signaling pathway remains to be explored. Thus, more solid and rescue assay are needed to elucidate its function on NSCs’ behavior. In addition to its physiological role, the function of Porf-2 on pathological conditions, such as optic nerve injury-based NSCs transplantation, has not been elucidated yet, which may provide us a new insight into targeting CNS repair.

The Wnt/β-catenin signaling pathway takes part in the proliferation of stem/progenitor cells and neural regeneration (Ming and Song, 2011; Zhang et al., 2011; Faigle and Song, 2013; Bengoa-Vergniory et al., 2014). Nuclear localization of β-catenin plays a critical role in the canonical Wnt signaling (Inoue et al., 2006). When Wnt signaling is activated, GSK-3β becomes inactivated, and β-catenin is stabilized, accumulates in the cytosol, and is translocated into the nucleus. In the nucleus, β-catenin complexes with T-cell factor/lymphoid enhancer factor-1 (TCF/LEF-1) and regulates proliferation-related gene expression to induce cell proliferation (Tiwari et al., 2014). Rac1, a member of small Rho GTPases, acts as a binary molecular switch and transduce upstream signals to downstream effectors by alternating between the “on” GTP-bound and the “off” GDP-bound states (Schmitz et al., 2000), which has been demonstrated to be involved in NSCs proliferation (Leone et al., 2010) and control the nuclear localization of β-catenin during canonical Wnt signaling in mouse embryonic limb bud ectoderm (Wu et al., 2008).

Here, we explored the effect of rat Porf-2 on NSCs proliferation and neuronal differentiation *in vitro* and in the retina. We develop a novel strategy for inhibiting primary NSCs proliferation via Porf-2. By using combined signal inhibitor and activators, we elucidated the molecular and cellular mechanisms by which Porf-2 exerts its effects. We show that Porf-2 interacts directly with Rac1, regulates the nuclear localization of β-catenin, and mediates NSCs proliferation. Additionally, by targeting Porf-2, the NSCs proliferation is increased and the visual function recovery is significantly improved in optic nerve injury rats.

## Experimental Procedures

### Isolation, Culture, and Identification of NSCs

During the experiments, animal handling was performed according to the guidelines of the Animal Care Committee of the Shanghai Jiao Tong University School of Medicine. The isolation, culture, and identification of NSCs were done according to our earlier published studies (Yang et al., 2014). Briefly, pregnant Sprague–Dawley rats (Shanghai Experimental Animal Center, Shanghai, China) were deeply anesthetized with a mixture of ketamine and xylazine (3:1 ratio) and carefully dissected to get embryos (embryonic day 14). The tissues were placed to cold phosphate-buffered saline (PBS), and hippocampus were carefully dissociated. The hippocampus cells were incubated in Dulbecco’s modified Eagle’s medium-F12 medium supplemented with 1% N2, 2% B27 supplement, 2 mmol/l glutamine, 20 ng/ml epidermal growth factor (EGF), 20 ng/ml basic fibroblast growth factor (bFGF), 100 U/ml penicillin, and 100 μg/ml streptomycin. EGF and bFGF were removed from the growth medium to induce differentiation, and the medium was supplemented with 1% fetal bovine serum (FBS). A single-cell suspension was prepared from the neurospheres by trypsinization, and then the cells were ready for subsequent study.

All the methods were in accordance with the approved guidelines and all the experimental protocols were approved and monitored by the Animal Care Committee of the Shanghai Jiao Tong University School of Medicine.

### Construction of the Recombinant Lentivirus and Lentiviral Transfection

To knock down or overexpress Porf-2, NSCs were transfected with Porf-2 shRNA lentiviruses or lentiviruses that overexpressed Porf-2, and the cells were subjected to puromycin selection. The procedure using lentiviral vectors has been approved by the Institutional Biosafety Committee of the Shanghai Jiao Tong University School of Medicine. The rat Porf-2 shRNA sequence: GCGATGGAACTCTGGTAATAA. The overexpression sequence are consistent with the cDNA of rat full-length Porf-2/Arghap39 in GenBank (NM_173122.3). Recombinant lentiviruses were produced by co-transfecting 293T cells with pHBLV-Porf-2 shRNA-U6-Zsgreen or pLenti-Porf-2-Ubc-EGFP-3FLAG expression plasmid and packaging plasmids. 293T cells were incubated in a 6-well plate with 6.25 × 10^5^ cells in 2 ml of culture medium per well 24 h before transfection. When the cells reached 70% confluence, 1 μg of pHBLV-Porf-2 shRNA-U6-Zsgreen or shRNA pHBLV-U6-Zsgreen, psPAX2, and pMD2.G mix (at a ratio of 2:1:1) and 6 μl of Fugene^®^6 transfection reagent were added into each well for knockdown co-transfection. For overexpression, pLenti-Porf-2-Ubc-EGFP-3FLAG or pLenti-Ubc-EGFP-3FLAG, PLP1, PLP2, and PLP/VSVG mix (at a ratio of 2:1:1:1) and 6 μl of Fugene^®^6 transfection reagent (Roche) were added. Cells were seeded at 37°C in a 5% CO_2_ incubator for 16–18 h and then re-fed with viral harvest medium at 1.5 ml per well. Viral particles were harvested 42 h post-transfection by collecting the medium and filtering through a 0.45-μm filter. The particles were ready for immediate transduction.

Neurospheres were dissociated into single cells before transduction, subcultured into 24-well plates at a concentration of 2–6 × 10^4^ cells/well, and grown in an incubator at 37°C with 5% CO_2_ in air for 24 h. The supernatant containing the pHBLV-Porf-2 shRNA-U6-Zsgreen or pLenti-Porf-2-Ubc-EGFP-3FLAG vector was added into the NSCs-containing wells at five times the multiplicity of infection (MOI = 5). After incubation for 8 h, the medium was replaced. Cells were observed to determine the efficiency of transfection. NSCs were infected with pHBLV-Porf-2 shRNA-U6-Zsgreen or pLenti-Ubc-EGFP-3FLAG vector using the same procedure. The surviving cells were ready for the assessment of Porf-2 expression levels and were seeded at a density of 1 × 10^5^ cells on PLL-coated coverslips in 24-well plates for the proliferation and differentiation of NSCs.

### Neurosphere Growth Kinetics Assay

In order to assess the effects of Porf-2 knockdown or overexpression on cell proliferation and neurosphere formation, the neurosphere growth kinetics assay was used. In brief, a single-cell suspension was incubated in a 12-well plate at a density of 50 000 cells/well in neurobasal medium containing B-27, N-2, bFGF, and EGF. The number and size of neurospheres in all the groups were taken under an Olympus CK-40 microscope (Olympus, Tokyo, Japan) and analyzed using NIS Elements BR imaging software.

### Treatment of NSCs with Rac1 or Wnt Pathway Activator or Inhibitor and Wnt3a Protein

After lentiviral transfection, the cultured primary hippocampal NSCs were then separately treated with fresh medium with a Rac1 activator (20 nmol/l CN04-A), Rac1 inhibitor (NSC23766, 10 mmol/l), Wnt activator (LiCl, 10 mmol/l), or β-catenin/TCF inhibitor (FH535), and Wnt3a (100 ng/ml) and were then incubated for 48 h. After treatment, a CCK-8 assay, EdU assay, immunocytochemistry, and Western blot analysis were performed to study NSCs proliferation and the protein levels of cyclin D1 and p21.

### Proliferation Assay

The proliferation of NSCs was determined using a CCK-8 assay, a sensitive non-radioactive colorimetric assay (Beyotime, China). NSCs were dissociated mechanically and seeded into 96-well plates (5 × 10^3^ cells/well), and cells were cultured with growth medium for different periods of time (1, 2, 3, 4, 5, 6, 7, 10, or 14 days). CCK-8 solution was added to the cell culture medium to a final concentration of 10 μl per 100 μl of medium and incubated at 37°C in 5% CO_2_ in air. Absorbance was measured 4 h later at a wavelength of 450 nm using a microplate reader (ELx800, BioTek Instruments Inc.).

Neural stem cells proliferation was also analyzed by EdU assay. EdU was added to the cultured NSCs 4 h before cell fixation. The EdU incorporated cells were proceed for immunofluorescence. Following a 20-min fixation with 4% paraformaldehyde/4% sucrose, the cells were washed and incubated with 0.5% Triton X-100/PBS for 30 min at room temperature (RT). The cells were permeabilized using the Click-iT reaction cocktail [1x with 87.5% (v/v) Click-iT Reaction Buffer, 2% (v/v) CuSO4, 0.05% (v/v) fluorescent azide (Alexa Fluor 647), and 10% (v/v) Reaction Buffer Additive]. The cells were washed and immunocytochemistry was conducted.

The number of EdU-positive cells were quantified in 8–10 randomly selected fields on each coverslip (for a total of 900–1,200 cells per coverslip), and the data were presented as the percentage of the total number of live GFP^+^ cells. At least three independent experiments was conducted for each condition.

### Immunoprecipitation

The collected cells were lysed in lysis buffer supplemented with protease inhibitors on ice for 15 min, and centrifugated at 14,000 ×*g* at 4°C for 10 min. The protein lysate was immunoprecipitated with an agarose-immobilized antibody (1 mg of porf-2 antibody as indicated or isotype control antibodies) overnight at 4°C.

### Rac1 Activation Assay

Rac1 activity was assessed using the Rac1 Activation Assay Kit (Millipore) according to the manufacturer’s instructions. Briefly, cell lysates were clarified by centrifugation at 14,000 ×*g* at 4°C for 10 min. Equal volumes of lysates were incubated with beads to pull down activated Rac1 proteins. After incubation at 4°C for 1 h, the beads were washed three times with cold MLB buffer. The Rac1 proteins were eluted with sample buffer and subjected to sodium dodecyl sulfate–polyacrylamide gel electrophoresis (SDS–PAGE). Western blot was analyzed using anti-Rac1 antibodies (Millipore). GAPDH was used as a housekeeping gene for normalization.

### Quantitative Real-Time PCR

Total RNA was isolated from transfected NSCs using TRIzol reagent, and genomic DNA was eliminated with RNase-free DNase. RNA pellets were re-suspended in DEPC-treated water. Quantitative real-time PCR was performed with an ABI PRISM 7700 sequence detection system (Applied Biosystems, United States). Reactions were done in duplicate with SYBR Green PCR Master mix (Applied Biosystems, United States). Forty cycles of PCR amplification were conducted according to the following program: primary extension of 94°C for 5 min; cycling at 94°C for 0.5 min, 55°C for 0.5 min, and 72°C for 0.5 min; and final extension at 72°C for 10 min. Amplification plots and cycle threshold values from the exponential phase of the PCR were analyzed using ABI Prism SDS1.7 software (Applied Biosystems). Expression of the cellular housekeeping gene β-actin is though to be a control to normalize values. Relative expression was quantified using the ΔΔCt method. The primer sequences are listed in **Table 1**.

**Table 1:** Primer sequences used for quantitative real-time PCR.

Gene name	Forward primer	Reverse primer
Porf-2	5′-CAAAGCAGCCACGCCATATC-3′	5′-TAAGAGGGATCAGGGCACGA-3′
β-actin	5′-CCCGCGAGTACAACCTTCT-3′	5′-CGTCATCCATGGCGAACT-3′
GSK-3β	5′-ATCAAGGCACATCCTTGGAC-3′	5′-ACGGCTACACAGTGCGATT-3′
Disheveled-1	5′-TCACGCTCAACATGGAGAGGCA-3′	5′-GCACGGCATCATCGTTGCTCAT3-3′
Frizzled-1	5′-CATCGCGTACAATCAGACCA-3′	5′-GCGTCCTCCTGATTCGTG-3′
β-Catenin	5′-GACCACAAGCAGAGTGCTGA-3′	5′-ACTCGGGTCTGTCAGGTGAG-3′
Wnt-3	5′-GCTACTCGGCCTCCTGCT-3′	5′-GGCCAGAGACGTGTACTGCT-3′
Wif1	5′-AGCCATTCCCGTCAATATCCA-3′	5′-ACCTATTTCCGTAGTACCGTCT-3′
Axin1	5′-AGGGCCCCCTCAAGTAGAC-3′	5′-CTCCCTCCAAGATCCATACCT-3′
Axin2	5′-GCAGGACCCACATCCTTCT-3′	5′-AGCCAGTCTCTTTGGCTCTTT-3′
CyclinD1	5′-GCACAACGCACTTTCTTTCC-3′	5′-TCCAGAAGGGCTTCAATCTG-3′
LRP-5	5′-CATCCATGCTGTGGAGGA-3′	5′-TGTCTCGGGCACAAGGAT-3′
LRP-6	5′-CATGATACGAAAGGCACAAGAA-3′	5′-TCTGATTTGGAACCGAGCTT-3′
Fzd-1	5′-CATCGCGTACAATCAGACCA-3′	5′-GCGTCCTCCTGATTCGTG-3′
Fzd-6	5′ -GAAAAGCAGCGTATCGGAAG-3′	5′-GGAACGCCCTTTGGACTTAC-3′
LEF1	5′-TGGTAAACGAGTCCGAAATCA-3′	5′-TGTGTTTGTCCGACCACCT-3′
APC	5′-CGTGGCCCAGTTAAAATCC-3′	5′-AAATTCCGCAAAACACTTGC-3′
Dkk-1	5′-CGGGAATTACTGCAAAAACG-3′	5′-CAATGATGCCTTCCTCGATT-3′
p21	5′-CTGCTCTCCCTTCCTCAGAC-3′	5′-TGAGGTAGGACCAGGAAACC-3′

### Western Blot

Samples were harvested at each time point into protein extraction buffer. Equal amounts of protein were denatured for 5 min at 95°C in sample buffer and separated by SDS–PAGE. Western blot analysis was performed using antibodies against Porf-2 (1:1,000; Millipore, United States), Wnt3a (1:500), Disheveled (1:1,000), Dkk-1 (1:1,000), Wif-1 (1:1,000), GSK-3β (1:1,000), p-GSK-3β (1:500), β-catenin (1:1,000), TCF (1:1,000), cyclin D1 (1:1,000), p21 (1:1,000), and β-actin (1:10,000) diluted in 10% horse serum in TBS-T buffer (0.2 M NaCl, 25 mM Tris, pH 7.5, and 0.5 ml/L Tween-20), followed by incubation with a horseradish peroxidase (HRP)-coupled mouse secondary antibody (1:10,000). Blots were re-probed with a β-actin antibody (BD Bioscience, United States). Signals were quantified with an image analyzer (UVtec, United Kingdom). Porf-2 and β-catenin signals were normalized by comparison with the corresponding β-actin signal of the samples. The data are presented as the percentages of the normalized control signal.

### Immunocytochemistry

The specimens were performed for 30 min at RT with 4% paraformaldehyde in 0.1 M phosphate buffer (pH 7.4) and then washed three times with 0.01 M PBS (pH 7.4). Next, the specimens were incubated in 0.5% Triton X-100 for 30 min at RT and followed three times with 0.01 M PBS. Non-specific binding was blocked with 5% normal goat serum, and then the plates were done overnight at 4°C with mouse primary antibodies. After the specimens by washed with PBS, a secondary antibody was incubated for 2 h at RT in the dark. The following antibodies were performed: anti-β-catenin (1:250; Millipore, United States) for nuclear localization and IgG-Cy3-linked goal anti-mouse secondary antibody (1:500; Beyotime, China). The specimens were washed again in PBS and then mounted in ProLong mounting medium (Invitrogen) containing DAPI. Preparations were photographed under a fluorescence microscope system (Leica, Wetzlar, Germany), and the captured images were done with Adobe Photoshop software.

### Rat Optic Nerve Crush Model

Adult male Sprague–Dawley rats weighing 200–250 g came from by Shanghai Experimental Animal Center, Shanghai, China. The optic nerve crush was performed as described previously (Feng et al., 2010). In brief, a lateral canthotomy was made in the superior orbital rim, and an incision of the conjunctiva was performed lateral to the cornea. After the separation of retractor bulb muscle, the optic nerve was exposed beneath the external ocular muscle. A Yasargil aneurysm clip (catalog No. FT495T, Aesculap AG & Co, Tuttlingen, Germany) was then placed 2 mm behind the posterior eye pole for 9 s.

All procedures were performed on the left eye under aseptic conditions. Twelve rats underwent unilateral sham optic nerve crush, in which the optic nerve was exposed and then the clip was placed 2 mm behind the posterior eye pole but removed immediately without closure.

### Subretinal Transplantation

All the cultured cells are dissociated before transplantation. NSCs were engrafted into the subretinal space immediately after the optic nerve crush with a transscleral approach as previous procedure (Yang et al., 2014). Briefly, under ophthalmic microscopic observation, the temporal conjunctiva was dissected at the limbus. A sclerotomy was done tangentially between the two vortex veins, approximately 1 mm beyond the limbus and directed toward the posterior pole, with a 33-gauge needle. A 33-gauge blunt needle attached to a 10-μl syringe (Hamilton, Reno, NV, United States) was introduced tangentially through the sclerotomy site into the subretinal region, indicating retinal detachment confirmed microscopically. Subsequently, the same procedure was repeated to slowly inject a suspension of GFP-expressing cells (2 μl contains 2.0 × 10^5^ cells) (GFP-NSCs, porf-2 overexpression NSCs, porf-2 knockdown NSCs, respectively). The duration of the injection lasted approximately 30 s, and the needle remained within the subretinal space for 2–3 min before slow withdrawn. In the control studies, 0.01 M PBS was injected into the subretinal space. At 14 days after transplantation, rats were sacrificed for assessing NSCs proliferation, and at 10 weeks after transplantation for assessing NSCs differentiation.

### Tissue Processing and Immunohistochemistry

Rat retina sections were prelabeled *in vivo* (intraperitoneal) with BrdU (50 mg/kg) for the final 24 h. To assess the influence of Porf-2 on proliferation and differentiation in the host retinas, rats were sacrificed at the indicated time. At these time points, rats were anesthetized with pentobarbital sodium (200 mg/kg) and perfused transcardially with 4% paraformaldehyde. After eyes enucleated, the anterior segments and the lens were removed, and the eyecups were post-fixed (free-floating) in 4% paraformaldehyde for an additional 2 h, washed with PBS, and transferred to 30% sucrose in PBS overnight for cryosectioning. The eyecups were embedded in optimal cutting temperature compound (OCT; Miles, Elkhart, IN, United States) after eyecup alignment so that the horizontal planes were parallel to the cutting plane. Next, 20-μm-thick frozen sections were cut with a cryostat. The sections were mounted onto clean, subbed slides and stored at -20°C until processing. Continuous sections including the injury site were cut for each eye.

For immunohistochemistry, retinal sections were incubated overnight at 4°C with anti-BrdU (1:500; Sigma, United States). The sections were incubated with fluorophore-tagged secondary antibodies.

### Cell Counts

Cell counting in the histological analysis was done as described previously (Yang et al., 2014). Fourteen days after transplantation, the eyes were proceeded for the analysis of proliferation, and for differentiation at 10 weeks after transplantation. Systematic random sampling was done to count the number of cells in the retina. To measure proliferated or differentiated NSCs in the injured retina, every sixth retina section (20 μm thickness), with a 120-μm intersection interval, was performed. Cell count was done in a blinded fashion under a fluorescence microscope. Double labeling of cells with GFP plus anti-BrdU or cellular differentiation markers was confirmed. The approximate number of proliferated or differentiated NSCs in each group was calculated by summing the cells in each section and then multiplying by 6.

### F-VEP Measurement

The flash visual evoked potentials (F-VEP) were assessed according to international standards of clinical visual electrophysiology with the visual electrophysiology system (Chongqing Medical Equipment Factory, China). Briefly, the anesthetized rats were placed to the experiment station. After pupils dilated with amide tropicamide, the recording electrode was subcutaneously inserted into the skin at the occipital protuberance, and the reference electrode into the skin at the midpoint of the eyes, and the ground electrode into the skin behind the right ear. After rats were allowed to adapt to the dark for 15 min, F-VEP was recorded at 10 weeks after transplantation. Three stable waveforms were recorded for each animal for both the injured and transplanted eyes. The recorded F-VEP waveform was a relatively stable N-P-N wave, named N1, P1, and N2. The latency of the initial N1, P1, and N2 waves was recorded in milliseconds (ms), and the amplitude of N1-P1 and P1-N2 was recorded in microvolts (μV). The peak latencies of N1 were performed from the beginning to the peak of N1. The peak latencies of P1 were measured from the beginning to the bottom of the P1 peak, etc. The amplitude value of N1-P1 was from the N1 peak to the bottom of P1. Each index was done three times and averaged.

### Statistical Analysis

All data are presented as the means ± SD. Significant differences among the groups were calculated with SPSS version 12.0 (Chicago, IL, United States) or GraphPad Prism version 4.0 (San Diego, CA, United States). An unpaired Student’s *t*-test or one-way ANOVA followed by Fisher’s least square difference (LSD) *post hoc* test was used for a statistical comparison of the mean values between two or three groups, respectively. A repeated-measures two-way ANOVA was used to compare matched data at multiple time points. *P*-values < 0.05 were considered statistically significant.

## Results

### Identification of NSCs and Stable Knockdown or Overexpression of Porf-2 in NSCs after Lentiviral Transfection

β-tubulin and GFAP staining of these cultures showed that cultured NSCs had a neural phenotype. The cells were positive for nestin and Porf-2 (**Supplementary Figure S1**).

**FIGURE 1:**
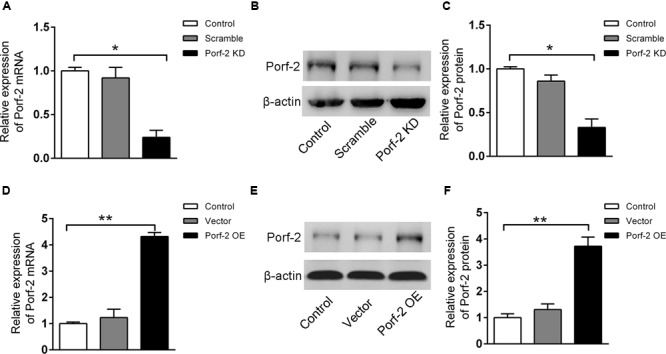
Stable knockdown or overexpression of Porf-2 in neural stem cells (NSCs) after lentiviral transfection. **(A)** Real-time PCR analysis of Porf-2 mRNA expression after Porf-2 knockdown in NSCs. Scramble, cells transfected with non-targeting shRNA as a negative control; KD, cells in which Porf-2 was knocked down by Porf-2-targeted shRNA. **(B)** The protein level of Porf-2 was detected by immunoblot after Porf-2 knockdown in NSCs. β-actin was used as an internal control. **(C)** Quantification of the band intensity of immunoblotting in **(B)**. The band density was normalized to β-actin signal in the same sample. **(D)** Real-time PCR analysis of Porf-2 mRNA expression after Porf-2 overexpression in NSCs. OE, overexpression. **(E)** The protein level of Porf-2 was detected by immunoblot after Porf-2 overexpression. β-actin was used as an internal control. **(F)** Quantification of the band intensity of immunoblotting in **(E)**. The band density was normalized to β-actin signal in the same sample. ^∗^ *p* < 0.05, ^∗∗^ *p* < 0.01 compared with the control group, *n* = 3.

To investigate the biochemical mechanisms of Porf-2 functions, we generated cultured NSCs that exhibited either stable knockdown or stable overexpression of endogenous Porf-2. We observed that Porf-2 mRNA (**Figure 1A**) and protein (**Figures 1B,C**) expression was significantly down-regulated after the transfection of Porf-2 shRNAs compared with control cells over several passages. Additionally, after transfection of the gene encoding Porf-2, the expression of Porf-2 mRNA (**Figure 1D**) and protein (**Figures 1E,F**) was up-regulated compared with control cells and vector cells (transfected with empty vector). These results demonstrated that Porf-2 was down-regulated and overexpressed via lentiviral transfection in cultured primary NSCs.

### Effect of Porf-2 Downregulation or Overexpression on NSCs Proliferation *In Vitro*

Neural stem cells growth and proliferation under conditions of Porf-2 knockdown or overexpression were measured by neurosphere growth kinetics assay, CCK-8 assay, and EdU assay.

**FIGURE 2:**
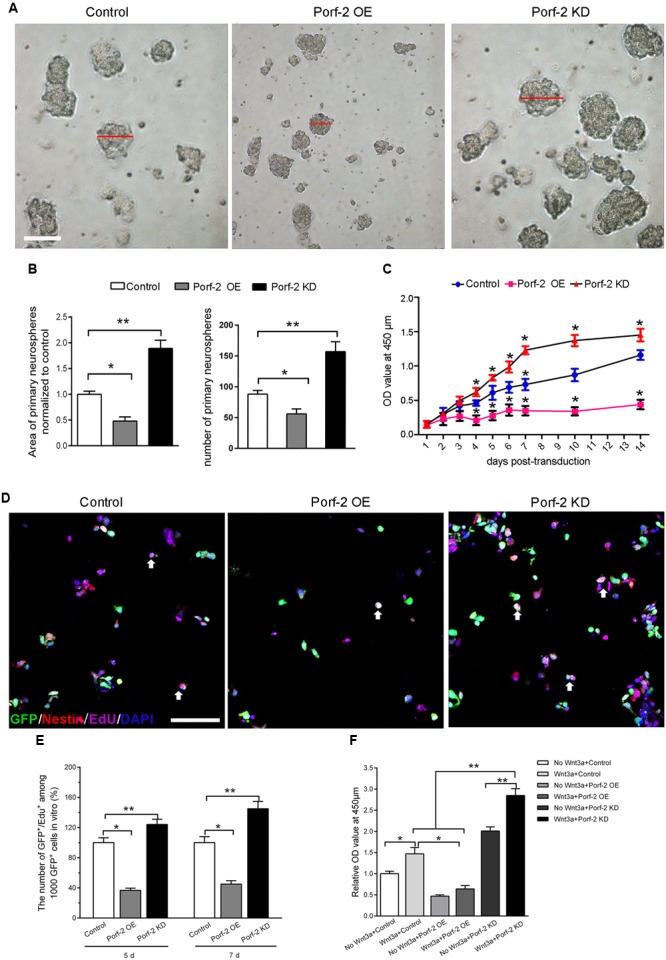
Porf-2 reduces growth/proliferation of hippocampus-derived NSCs. **(A)** Representative images of NSC neurospheres in Control, Porf-2 KD, and Porf-2 OE groups. The red line indicated the diameter of the neurosphere. Scale bar: 100 μm. **(B)** Quantification of the area and the number of neurospheres in each group. The data was normalized to the Control group. Values are expressed as the mean ± SD (*n* = 3). ^∗^ *p* < 0.05, ^∗∗^ *p* < 0.01. **(C)** CCK-8 assays were performed to analyze the growth/proliferation of NSCs in different groups. Significant changes are shown as ^∗^ *p* < 0.05 vs. the control group. **(D)** Porf-2 knockdown NSCs, Porf-2-overexpression NSCs, and GFP-NSCs were seeded into 96-well culture plates 5, 7 days after transduction. The white arrow indicates the EdU^+^GFP^+^ neural stem cells. The EdU assay was performed in each group. Scale bar: 100 μm. **(E)** The relative proliferation ratios (EdU^+^GFP^+^/GFP^+^) were calculated and normalized to control group (GFP-NSCs). **(F)** CCK8 assay showed the effect of Wnt3a protein on Porf-2 knockdown and Porf-2-overexpression-NSCs proliferation. Values are expressed as mean ± SD (*n* = 3). ^∗^ *p* < 0.05, ^∗∗^ *p* < 0.01.

In order to assess whether Porf-2 knockdown or overexpression show any effect on the number of NSCs, a neurosphere formation assay was performed. Neurospheres are free-floating spherical clusters of NSCs formed in the presence of specific mitotic growth factors. The number of clonal neurospheres *in vitro* is the measure of absolute putative stem cells. Gross morphology, size, and the number of neurospheres in each group were assessed (**Figures 2A,B**). The number and size of primary clonal neurospheres in Porf-2 knockdown group significantly increased as compared to control group and Porf-2 overexpression group (**Figures 2A,B**). Porf-2 overexpression decreased the number and size of neurospheres (**Figures 2A,B**). These results suggest that Porf-2 knockdown increases the number of multipotent NSCs and hence neurosphere formation. In addition, the CCK-8 assay showed that Porf-2 knockdown significantly increased NSCs growth on days 4, 5, 6, 7, 10, and 14 post-transduction among the three groups (**Figure 2C**), whereas Porf-2 overexpression led to reduced NSCs growth, indicating that Porf-2 expression negatively regulated growth.

Moreover, we further used the EdU incorporation assay to further evaluate the effect of Porf-2 knockdown or overexpression on NSCs proliferation (**Figures 2D,E**). On days 5 and 7 post-transduction, cells received 10 μM EdU for 30 min. The incorporated EdU was detected using an anti-EdU kit. The relative ratio of EdU^+^ cells increased by approximately 50% in the Porf-2 knockdown group compared with the control group (^∗∗^*P* < 0.01) and decreased by 40% in the Porf-2 overexpression group compared with the control group (**Figure 2E**). These results showed that the downregulation of Porf-2 promoted NSCs proliferation, whereas its overexpression attenuated NSCs proliferation.

### Effect of Wnt3a Protein on Porf-2 Downregulation or Overexpression Mediated-NSCs Proliferation *In Vitro*

In the presence of Wnt3a protein, NSCs grow faster in Porf-2 knockdown group compared with control group and Porf-2 overexpression group showed by CCK8 assay (**Figure 2F**). Moreover, Porf-2 overexpression is enough to reverse the effect of Wnt3a on NSCs proliferation showed by Wnt3a+control group and Wnt3a+Porf-2 overexpression group (**Figure 2F**).

### Alteration of Rac1 Activity and the Wnt/β-Catenin Signaling Pathway in Porf-2 Knockdown or Overexpression NSCs

**FIGURE 3:**
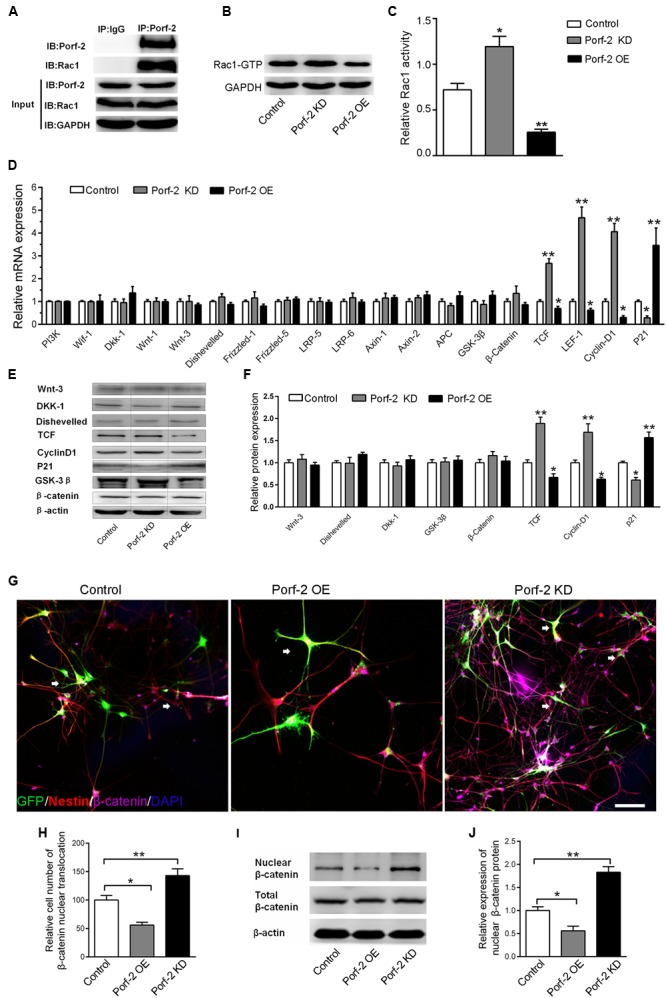
Effect of Porf-2 downregulation or overexpression on Rac1 activity and Wnt/β-catenin signaling pathway in NSCs. **(A)** Co-immunoprecipitation experiments showed that Porf-2 and Rac1 co-immunoprecipitated. **(B,C)** Cell lysates were processed to analyze Rac1 activation by pull-down. Effect of Porf-2 knockdown or overexpression on Rac1 activation. NSCs were transfected with knockdown, overexpression, or control vector. GAPDH was used as a housekeeping gene for normalization. **(D)** RT-PCR analysis was performed to determine the relative mRNA expression of Wnt/β-catenin pathway genes in the three groups.*β-actin was used as a housekeeping gene for normalization. **(E,F)** Western blot analysis suggested that Porf-2 knockdown significantly increased the relative levels of T-cell factor (TCF) and cyclin D1 and decreased the levels of the inhibitory protein p21, whereas Porf-2 overexpression significantly decreased the relative levels of TCF and cyclin D1 and enhanced the levels of the inhibitory protein p21. **(G)** Immunofluorescence photomicrograph showing nuclear translocation of β-catenin, a key regulatory protein of the Wnt pathway (purple), with the NSCs marker nestin (red) in the three groups. The white arrows the expression of β-catenin in GFP^+^ cells. Scale bar: 100 μm. **(H)** Quantify the ratio of the cell number of nuclear translocation of β-catenin in GFP^+^ Nestin^+^ cells. The ratio was normalized to control group. Quantitative analysis suggested that Porf-2 downregulation significantly increased the nuclear translocation of β-catenin, whereas Porf-2 overexpression significantly reduced the nuclear translocation of β-catenin. **(I)** Western blot analysis of the expression of nuclear β-catenin in the three groups. **(J)** The relative expression levels of nuclear β-catenin are represented by the ratio of nuclear β-catenin in the three groups. Values are expressed as the mean ± SD (*n* = 3 per group). ^∗^*p* < 0.05, ^∗∗^*p* < 0.01 vs. control. Scale bar = 100 μm.*

Co-immunoprecipitation experiments showed that Porf-2 and Rac1 co-immunoprecipitated; thus Porf-2 and Rac1 appear to interact directly (**Figure 3A**). To assess the effects of Porf-2 on Rac1 activity, we constructed Porf-2 knockdown or overexpression NSCs and found that Porf-2 downregulation provoked an increase in Rac1 activity, whereas Porf-2 overexpression induced a decrease in Rac1 activity (**Figures 3B,C**). This result indicated that Porf-2 is a negative regulator of Rac1 activity.

Next, we assessed the effects of Porf-2 on the levels of gene and protein expression in the Wnt/β-catenin pathway in Porf-2-overexpression and down-regulated NSCs. Porf-2 knockdown significantly increased the expression of genes such as nuclear transcription factor (LEF-1, TCF) and the Wnt target gene cyclin D1, and decreased expression of the proliferation-associated gene p21 (**Figure 3D**). These effects were reversed by Porf-2 overexpression (**Figure 3D**). The mRNA levels of Wif-1, Dkk-1, Wnt-1, Wnt-3, Disheveled, Frizzled-1, Frizzled-5, LRP-5, LRP-6, and the β-catenin destruction complex (β-catenin, Axin-1, Axin-2, APC, and GSK-3β) were not significantly different after Porf-2 knockdown or overexpression (*P* > 0.05) (**Figure 3D**). Immunoblot analysis revealed that Porf-2 knockdown significantly enhanced the protein levels of TCF, and cyclin D1 and reduced p21 levels (**Figures 3E,F**). In contrast, Porf-2 overexpression significantly reduced the protein levels of TCF and cyclin D1 and increased p21 levels (*P* < 0.05, **Figures 3E,F**). Levels of Wnt-3, Dkk-1, Disheveled, and β-catenin were not significantly different after Porf-2 knockdown or overexpression (*P* > 0.05, **Figures 3E,F**).

Nuclear translocation of β-catenin, a key regulatory protein of the Wnt pathway, was enhanced in the Porf-2 knockdown group compared with the control group but decreased in the Porf-2 overexpression group (**Figures 3G,H**). Western blot analysis of the expression of nuclear β-catenin showed that, compared with the control group, the relative expression levels of nuclear β-catenin were higher in the Porf-2 knockdown group but lower in the Porf-2 overexpression group (**Figures 3I,J**).

### Effect of Porf-2 Knockdown or Overexpression on NSCs Proliferation via the Rac1-Wnt/β-Catenin Pathway

**FIGURE 4:**
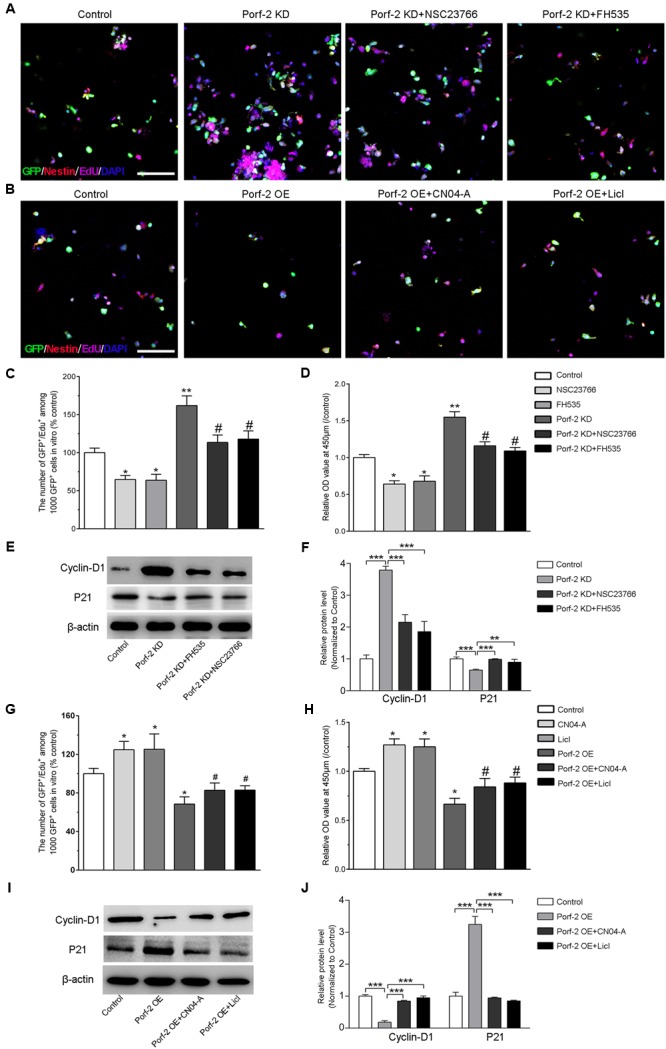
Effect of Porf-2 knockdown or overexpression on NSCs proliferation via the Rac1-Wnt/β-catenin pathway. **(A,B)** Representative photographs of proliferation of EdU-positive NSCs. Porf-2 knockdown and Porf-2-overexpression NSCs were treated with a Rac1 activator (CN04-A), Rac1 inhibitor (NSC23766), Wnt activator (LiCl), or β-catenin/TCF inhibitor (FH535). Scale bar: 100 μm. **(C,D,G,H)** The relative proliferation ratio of EdU-positive NSCs and CCK8 assay. The relative proliferation ratios are represented in Porf-2 knockdown and Porf-2-overexpression NSCs, which were treated with a Rac1 activator (CN04-A), Rac1 inhibitor (NSC23766), GSK-3β inhibitor (LiCl), or β-catenin/TCF inhibitor (FH535). **(C,D)** ^∗^ *p* < 0.05, ^∗∗^ *p* < 0.01 vs. control. # *p* < 0.05 vs. Porf-2 KD. **(G,H)** ^∗^*p* < 0.05 vs. control. # *p* < 0.05 vs. Porf-2 OE. **(E,F,I,J)** The effect of activators or inhibitors of Rac1 or the Wnt/β-catenin signaling pathway on Porf-2 knockdown or overexpression NSCs growth and proliferation-associated proteins. ^∗∗^*p* < 0.01, ^∗∗∗^*p* < 0.001.

Next, to study the effects of Porf-2 downregulation or overexpression on NSCs proliferation via the Rac1-Wnt/β-catenin pathway, NSCs cultures were treated with a Rac1 activator (CN04-A), Rac1 inhibitor (NSC23766), GSK-3β inhibitor (LiCl), or β-catenin/TCF inhibitor (FH535) (**Figure 4**).

We confirmed that Porf-2 contains a Rac1GAP domain, which can negatively regulate the activity of Rac1 (**Figures 3A,B**). To study the role of Rac1 in Porf-2-mediated NSCs proliferation, we first determined whether Rac1 activation and inactivation could affect NSCs proliferation with Porf-2 knockdown or overexpression utilizing a Rac1 inhibitor (NSC23766) or Rac1 activator (CN04-A).

We found that Porf-2 downregulation significantly promoted the growth and proliferation of NSCs in culture (**Figures 2, 4C,D**), whereas Porf-2 overexpression led to reduced growth and proliferation (**Figures 2, 4G,H**). Porf-2 knockdown NSCs were treated with the Rac1 inhibitor (NSC23766), and Porf-2-overexpression NSCs were treated with the Rac1 activator (CN04-A). The treated NSCs were evaluated using CCK-8 and EdU assays (**Figure 4**). The growth and proliferative activities induced by Porf-2 knockdown were significantly reduced in cells treated with the Rac1 inhibitor (NSC23766) (**Figures 4A,C,D**, #*P* < 0.05 vs. Porf-2 KD), whereas the anti-growth and proliferative activities induced by Porf-2 overexpression were significantly reduced in cells treated with the Rac1 activator (CN04-A) (**Figures 4B,G,H**, #*P* < 0.05 vs. Porf-2 OE). Moreover, cyclin D1 protein levels were significantly decreased, and p21 levels increased in Porf-2 knockdown NSCs cultures treated with NSC23766 (**Figures 4E,F**), suggesting decreased NSCs proliferation. Treatment with the Rac1 activator (CN04-A) significantly enhanced the expression of cyclin D1 and reduced the expression of p21 in Porf-2-overexpression NSCs cultures (**Figures 4I,J**), suggesting increased NSCs proliferation. Together, Porf-2 exerted its inhibitory role of NSCs proliferation through Rac1, which is enough to reverse Porf-2’s function.

Similarly, the role of the Wnt/β-catenin pathway in Porf-2 knockdown or overexpression NSCs was elucidated by the CCK-8 and EdU assay (**Figure 4**). Interestingly, the proliferation-enhancing potential of Porf-2 downregulation was significantly blocked by Wnt pathway inhibitors, such as FH535, suggesting an alteration of the Wnt pathway due to Porf-2 knockdown (**Figures 4A,C,D**, #*p* < 0.05 vs. Porf-2 KD). Wnt pathway activators, such as LiCl, significantly enhanced cell proliferation and significantly reversed the inhibitory effect of Porf-2 overexpression (**Figures 4B,G,H**, #*P* < 0.05 vs. Porf-2 OE). These findings indicated that the Wnt/β-catenin pathway is involved in Porf-2-mediated alteration of NSCs proliferation. Moreover, cyclin D1 protein levels were significantly decreased and p21 levels increased in Porf-2 knockdown NSCs cultures treated with FH535, suggesting decreased NSCs proliferation (**Figures 4E,F**). Treatment with the Wnt pathway activator LiCl significantly enhanced the expression of cyclin D1 and reduced p21 in Porf-2-overexpression NSCs cultures, suggesting increased NSCs proliferation (**Figures 4I,J**). These results revealed that Porf-2 can regulate NSCs proliferation through the Wnt/β-catenin signaling.

### Increase of Wnt3a Protein Expression after Optic Nerve Crush

**FIGURE 5:**
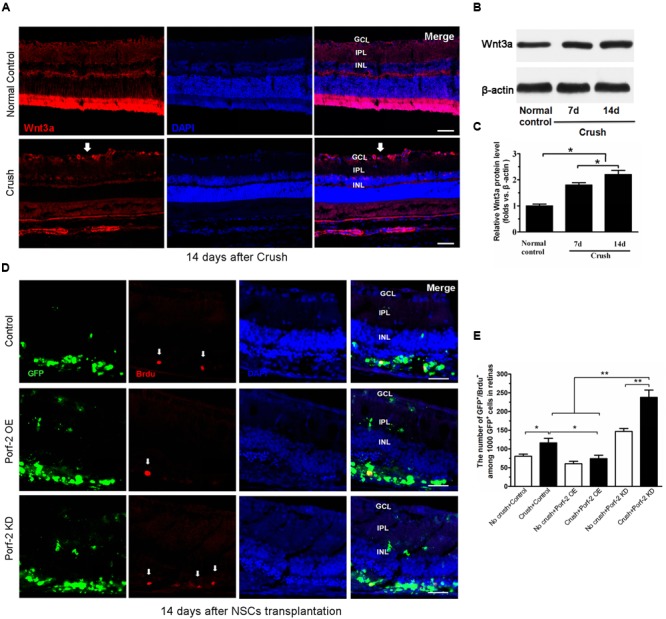
BrdU incorporation and Wnt3a expression after optic nerve injury. **(A)** The expression of Wnt3a protein in retina after optic nerve injury. Western blot analysis of Wnt3a protein in retina. Scale bars: 50 μm. Significant changes are shown as *p* < 0.05, ^∗^: vs. the control group. GCL, ganglion cell layer; IPL, inner plexiform layer; INL, inner nuclear layer. Immunofluorescence photomicrographs show the increase of Wnt3a protein in retina after optic nerve injury (white arrow indicated the Wnt3a expression in GCL). **(B)** Values were normalized to β-actin, used as a loading control. **(C)** Quantification of relative protein density after normalization with β-actin. **(D)** Characterization of BrdU-labeled cells at 14 days after transplantation in the control group, in the Porf-2-overexpression group and the Porf-2 knockdown group. White arrow indicates BrdU-positive cells. NSCs were transplanted into the subretinal space immediately after the optic nerve crush. Scale bars: 100 μm. **(E)** Comparison of proliferative capacity of control, Porf-2 knockdown NSCs, and Porf-2-overexpression NSCs at 14 days after transplantation (*n* = 6 rats). The ratio of each lineage is indicated on the graph. ^∗^*p* < 0.05, ^∗∗^*p* < 0.01. Values are expressed as the mean ± SD (*n* = 3 cultures).

To assess the expression of Wnt3a protein after optic nerve crush injury, western blot was performed. Immunofluorescence analysis revealed that the retinas from the normal control groups showed faint staining for Wnt3a along the inner border of the retina above the GCL. Compared with the normal control groups, the retinas from the operated rats exhibited an increase in Wnt3a staining (**Figure 5A**). The Wnt3a protein levels significantly increased in the retina after optic nerve injury, compared with the normal control animals (*P* < 0.05) (**Figures 5B,C**).

### Effect of Porf-2 Downregulation or Overexpression on the Proliferation of NSCs *In Vivo*

To assess the effect of Porf-2 downregulation or overexpression on NSCs proliferation at 14 days after transplantation *in vivo*, we transplanted Porf-2 knockout or overexpression NSCs into the subretinal space after optic nerve injury. The labeling index for GFP^+^/BrdU^+^ cells was measured in retinal sections for BrdU and DAPI staining (**Figures 5D,E**). After administration BrdU 48 h, we analyzed the percentage of BrdU^+^ cells in 1,000 randomly selected GFP^+^ cells under the same observation conditions. Porf-2 knockdown significantly increased the BrdU index of the retinas in comparison with control group (237.90 ± 19.33 in Porf-2 KD group vs. 116.46 ± 8.82 in control group. ^∗∗^*p* < 0.01; **Figure 5E**), whereas Porf-2 overexpression decreased compared to control (74.36 ± 8.05 in Porf-2 OE group vs. 116.46 ± 8.82 in control group. ^∗^*P* < 0.05; **Figure 5E**). Taking the *in vitro* data into consideration, we can conclude that Porf-2 knockdown significantly increase NSCs proliferation *in vivo* and *in vitro*. In addition, we found that no tumor-like cells aggregation at 10 week after transplantation, which destroyed the structure of retinas (data not shown).

### Effect of Porf-2 Knockdown or Overexpression on the Neuronal and Astrocytic Differentiation of NSCs *In Vitro* and *In Vivo*

**FIGURE 6:**
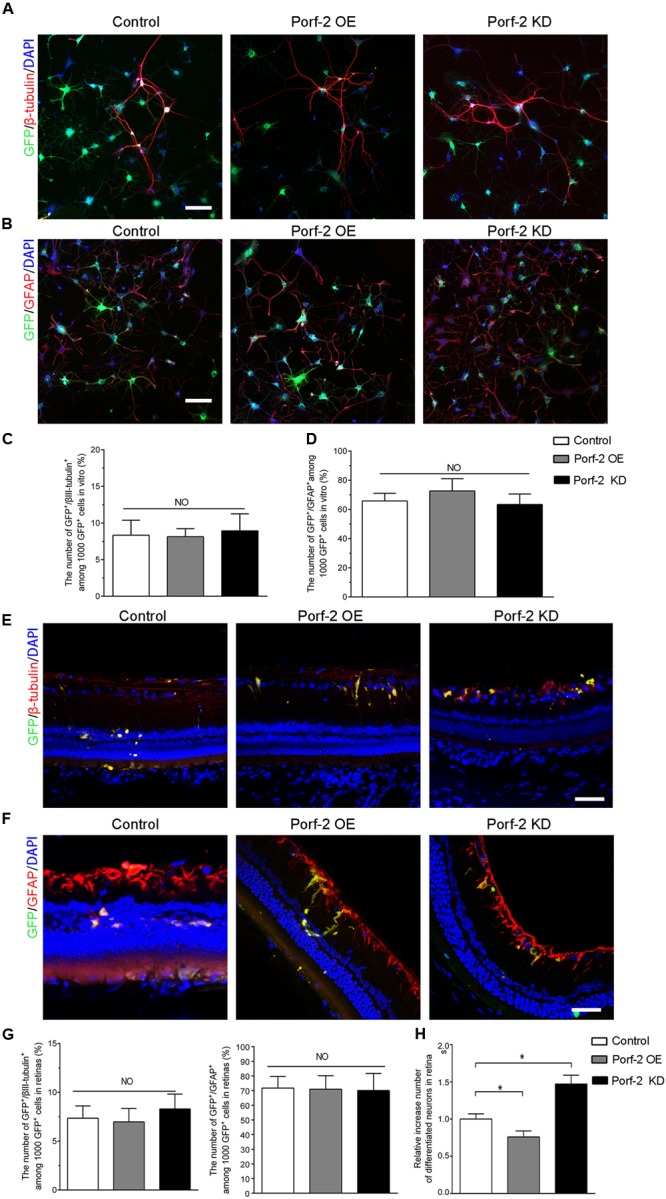
Effect of Porf-2 knockdown or overexpression on the neuronal and astrocytic differentiation of NSCs *in vitro* and *in vivo*. **(A,B)** Immunocytochemical analysis of Porf-2 knockdown and Porf-2-overexpression NSCs *in vitro*. Expression of β-tubulin III or GFAP in Porf-2 knockdown cells and Porf-2-overexpression cells after the induction of differentiation by serum starvation. Scale bars: 100 μm. **(C,D)** The percentage of β-tubulin III or GFAP double-positive cells was calculated. **(E,F)** Immunocytochemical analysis of Porf-2 knockdown and Porf-2-overexpression NSCs *in vivo* (*n* = 6 rats). Expression of β-tubulin III or GFAP in Porf-2 knockdown cells and Porf-2-overexpression cells at 10 weeks post-transplantation. Scale bars: 50 μm. **(G)** The percentage of β-tubulin III or GFAP double-positive cells was calculated. **(H)** Comparison the number of differentiated neurons in Control, Porf-2 KD and Porf-2 OE groups at 10 week after transplantation (*n* = 6). The data was normalized to Control group. ^∗^*p* < 0.05. Error bars indicate the SD of the mean.

After the induction of differentiation by serum starvation, 7.92 ± 2.32% of knockdown cells and 8.13 ± 1.10% of overexpression cells expressed the neuronal marker β-tubulin III compared with 8.33 ± 2.64% of control cells (**Figures 6A,C**). Furthermore, 69.38 ± 3.14% of knockdown cells and 72.66 ± 8.36% of overexpression cells expressed GFAP, a marker for astrocytes, compared with 65.81 ± 5.29% of control cells (**Figures 6B,D**). There was no significant difference in the neuronal and astrocytic differentiation of NSCs among the Porf-2 knockdown NSCs, Porf-2 overexpression NSCs and control NSCs.

To investigate the role of Porf-2 in the neuronal differentiation of NSCs *in vivo*, we transplanted Lenti-Porf-2-infected NSCs into the subretinal space of rats following optic nerve crush and examined the percentage of GFP^+^/β-tubulin III^+^ or GFP^+^/GFAP^+^ cells generated. Immunocytochemical staining (**Figures 6E–G**) revealed no significant difference in the percentage of GFP^+^/β-tubulin III^+^ cells (6.90% ± 1.52%, *n* = 6) in the Porf-2 knockdown (6.98% ± 1.37%, *n* = 6) or Porf-2 overexpression groups compared with the GFP group (7.35% ± 1.26%) (**Figures 6E,G**). However, the number of differentiation of NSCs into neurons of Porf-2 knockdown NSCs significantly increased than control or Porf-2-overexpression NSCs at 10 week after transplantation (**Figure 6H**). Immunocytochemical staining revealed no significant difference in the percentage of GFP^+^/GFAP^+^ cells (72.12% ± 11.64%, *n* = 6) in the Porf-2 knockdown (70.96% ± 9.21%, *n* = 6) or Porf-2 overexpression groups compared with the GFP group (71.77% ± 7.93%) (**Figures 6F,G**).

### Effect of Porf-2 Downregulation or Overexpression on F-VEP after Transplantation

**FIGURE 7:**
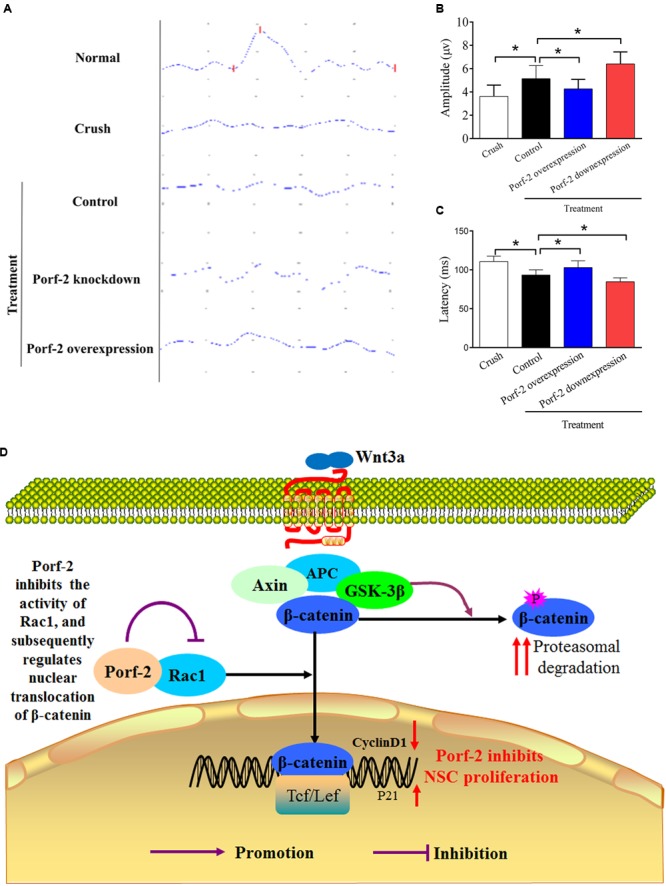
Evaluation of the recovery of the injured optic nerve by NPN wave pattern after transplantation. **(A)** Representative flash visual evoked potentials (F-VEP) tracings at 10 weeks after optic nerve crush injury in Crush, Porf-2-overexpression, Porf-2 knockdown and Control (GFP-NSCs) groups. **(B)** Bar charts showing F-VEP latency (*n* = 6 rats). **(C)** Bar charts showing F-VEP amplitude (*n* = 6 rats), ^∗^*p* < 0.05. **(D)** Exploration of the mechanism by which Porf-2 exerts its anti-proliferative effects on hippocampus-derived NSCs. Briefly, a direct interaction between Porf-2 and Rac1 reduces the activity of Rac1, which induces the nuclear translocation of β-catenin. β-catenin in the nucleus subsequently activates nuclear transcription factors and inactivates inhibitory factors, thereby resulting in the phosphorylation of many target proteins and finally regulating the proliferation of NSCs. P (red or purple): different sites of phosphorylation.

Compared with the control group (Crush + GFP-NSCs), the Porf-2 knockdown group showed significantly higher amplitudes of N1-P1 and P1-N2 in the Porf-2 knockdown group compared with the GFP-NSCs group, but lower amplitudes in Porf-2-overexpression group (**Figures 7A,B**). The peak latencies of the N1, P1, and N2 waves is shorter in the Porf-2 knockdown group compared with the GFP-NSCs group, but longer N1, P1, and N2 waves were observed in the Porf-2-overexpression group at 10 weeks post-transplantation (**Figures 7A,C**). The data suggested the Porf-2 knockdown NSCs transplantation may contribute to better F-VEP recovery after optic nerve injury.

## Discussion

Damage to the optic nerve leads to the loss of RGCs, subsequently causing reduced visual function and even blindness. NSCs pose multipotent capacity and differentiate into neurons, astrocytes, and oligodendrocytes (Ming and Song, 2011; Bond et al., 2015). Replacing injured RGCs is one of the promising approaches to the treatment of optic nerve injury, whereas a poor proliferation rate has limited the practical use of NSCs-based replacement therapy (Carreira et al., 2014). Numerous studies demonstrated that Wnt signaling plays a pivotal role in NSCs proliferation (Ming and Song, 2011; Faigle and Song, 2013). Previously, we also showed that overexpression of Wnt3a in NSCs induced activation of Wnt signaling and promoted proliferation both *in vitro* and *in optic* nerve injury, which suggests that targeting the Wnt3 signal may potentiate the therapeutic benefits of NSCs-based therapy in optic nerve injury (Yang et al., 2014). However, how the Wnt signaling exerts its role and whether the activation of Wnt signaling is regulated by other factors is unclear.

As a growth regulator, Porf-2 is expressed in rapid cell division tissue such as skin, the placental growth cone, and dividing testicular germ cells (Nowak and Gore, 1999). Porf-2 is most highly expressed in the hypothalamus, hippocampus, cerebral cortex, and anterior pituitary of neonatal and early postnatal rats (Nowak and Gore, 1999). However, the function of Porf-2 remains unknown for a long time. Recently, Ma and Nowak (2011) reported that Porf-2 knockdown increases proliferation and decreases drug-induced apoptosis in C17.2 cells. But there are lots of limitation and unsolved problem in this report (Ma and Nowak, 2011). Instead of using primary stem cell derived from the brain, an immortalized and artificially modified NSC line C17.2 was used, which have been reported to have different gene expression profiles and unique properties compared to primary NSCs (Martinez-Serrano and Bjorklund, 1997; Mi et al., 2005; Magnitsky et al., 2008). Furthermore, the results are based on a drug pre-treatment, but not a physiological condition (Ma and Nowak, 2011). Importantly, the Porf-2 described in Ma et al.’s study is not a full-length protein but a truncated transcript (Protein molecular weight: 65 kD) (Ma and Nowak, 2011). Until now, this is the only report describing a truncated transcript of Porf-2 (Ma and Nowak, 2011) and there is no more follow-up reports to confirm that yet.

Since a truncated transcript of Porf-2 may play a different role with a full-length one due to the different amino acid sequences, domains or structures. To uncover the role of Porf-2, we have constructed a mouse full-length Porf-2 previously (Protein molecular weight: about 130 kD) (Huang et al., 2016). Our previous study first showed that Porf-2 played an inhibitory role in NSCs proliferation, and the GAP domain is necessary for its function (Huang et al., 2016). It should be noted that in our previous study, in order to fully investigate the functional domain of Porf-2, we constructed three domain deleted Porf-2 plasmids (Huang et al., 2016). Different to the truncated transcript of Porf-2 reported by Ma and Nowak (2011), which is detected in endogenous C17.2 cell line, our truncated versions of Porf-2 are artificial mutations based on the amino acid and domain sequence of full-length Porf-2 (Huang et al., 2016). Moreover, we screened the possible pathway in mediating NSCs proliferation and found that Porf-2 mediated NSCs proliferation through the classical Wnt/β-catenin pathway (Huang et al., 2016). However, how Porf-2 functions through Wnt are still not fully validated. Here, we provide solid rescue assay by using Rac1 activator (CN04-A), Rac1 inhibitor (NSC23766), GSK-3β inhibitor (LiCl), or β-catenin/TCF inhibitor (FH535). Moreover, Porf-2 is expressed in the CNS in a wide range of species, including human, rat, mouse, zebrafish, and sheep (Nowak, 2003). We also present evidence that Porf-2 exhibits similar role in rat, suggesting its conserved function in vertebrate. In addition to physiological role, here we also show that after optic nerve injury, the Wnt signaling is activated. But the poor proliferation of NSCs in optic nerve injury may be due to the inhibitory role of Porf-2, since Porf-2 overexpression is enough to block the role of Wnt3a and Porf-2 knockdown significantly increased the NSCs proliferation in retina after optic nerve injury. This may provide us a new insight into targeting Porf-2 in CNS diseases.

To explore the effects of Porf-2 on NSCs proliferation, we established cell models in which Porf-2 was either knocked down or overexpressed by transfecting a Porf-2 shRNA or Porf-2 vector into NSCs, respectively. By using CCK-8 and EdU assays, we found that Porf-2 knockdown promoted NSCs proliferation, whereas Porf-2 overexpression decreased NSCs growth and proliferation. We microinjected Porf-2-overexpression NSCs or Porf-2 knockdown NSCs into the subretinal space after rat optic nerve crush injury, and NSCs proliferation increased with Porf-2 knockdown and decreased with Porf-2 overexpression. Moreover, transplantation of Porf-2 knockdown NSCs significantly improved visual function recovery compared with Porf-2-overexpression NSCs. Our study demonstrates the enhanced proliferative ability of Porf-2 knockdown NSCs in the injured retina, which indicates the potential advantages of combination therapies in treating CNS injury. The enhanced proliferative ability of NSCs may partially overcome the harmful factors of specific acute inflammatory activation. Importantly, the enhanced proliferative ability of NSCs could partially counteract the effects of the chronic CNS scar tissue that forms after CNS injury.

Notably, our results elucidate the underlying mechanism of the effect of Porf-2 on NSCs proliferation via the Rac1-Wnt/β-catenin pathway. Porf-2 downregulation activated Rac1, promoted the nuclear translocation of β-catenin and increased proliferation, and the effect was reversed by Porf-2 overexpression. β-catenin is known as the crucial effector in the Wnt signaling pathway and mediates several cellular processes, including cell proliferation (Gan et al., 2008). Wnt stimulation induces the β-catenin nuclear translocation, which triggers the transcriptional expression of target genes that function during cell proliferation. The activated Wnt pathway was assessed by immunocytochemistry to identify the subcellular localization of β-catenin (Inoue et al., 2006). β-catenin is a coactivator of TCF/LEF-dependent transcription. The translocation of stabilized β-catenin into the nucleus indicates the activation of Wnt signaling (Clevers and Nusse, 2012; Jamieson et al., 2012). In our study, Porf-2 downregulation induced the translocation of more β-catenin into the nucleus in NSCs than in the control group, which had more translocation than the Porf-2 overexpression group. However, we failed to find an increase in β-catenin protein expression in the cytosol in the Porf-2 knockdown or Porf-2 overexpression groups. In our study, GSK3β mRNA and protein levels did not change. The results are in good agreement with a previous study that showed that Rac1 mutation had no effect on β-catenin stabilization in the cytoplasm but abolished β-catenin nucleus accumulation (Wu et al., 2008). β-catenin in the cytosol is normally phosphorylated by GSK3β and is quickly degraded. Rac1 activates JNK1/2, phosphorylates β-catenin on Ser191 and Ser605, and subsequently induces the nuclear localization of β-catenin.

In the canonical Wnt signaling pathway, the β-catenin nuclear localization triggers the interaction with the TCF/LEF family of transcription factors, which induces the expression of Wnt pathway target genes such as cyclin-D1, suppresses p21, and enhances proliferation (Tiwari et al., 2014). To further clarify the role of downstream molecules of Porf-2, we used a Rac1 inhibitor and activator and a Wnt signaling pathway inhibitor and activator to treat knockdown and overexpression NSCs. We found that Porf-2 knockdown led to the activation of Rac1 and promoted the nuclear translocation of β-catenin in NSCs, as determined by a Rac GTPase activity assay and Western blotting. However, Porf-2 overexpression led to the inactivation of Rac1 and reduced the nuclear translocation of β-catenin in the Wnt pathway. The mRNA levels of the Rac1-Wnt pathway, the protein levels of cyclin D1 and activated Rac1 increased in Porf-2 knockdown NSCs, whereas decreased in Porf-2-overexpression NSCs. The CCK-8 and EdU assays showed that proliferation was reduced in Porf-2-overexpression NSCs, and this effect was reversed by an activator of Rac1 or the Wnt signaling pathway. NSCs proliferation was enhanced upon Porf-2 knockdown, which was attenuated by an inhibitor of Rac1 or the Wnt signaling pathway (NSC23766 and FH535, respectively). Western blotting revealed that Porf-2 overexpression decreased the cyclin D1 levels and increased P21 levels, which were reversed by activators of Rac1 or the Wnt signaling pathway (PDGF and LiCl, respectively). Conversely, Porf-2 knockdown increased the cyclin D1 levels and decreased P21 levels, which were attenuated by inhibitors of Rac1 or the Wnt signaling pathway. These findings reinforce the notion that the Rac1-Wnt signaling pathway is required for Porf-2 to regulate NSCs proliferation. Porf-2 knockdown significantly increased Wnt3a-induced NSCs proliferation, while Porf-2 overexpression significantly decreased Wnt3a-induced NSCs proliferation, indicating Wnt signal pathway was regulated by Porf-2. After optic nerve injury, the Wnt3a is up-regulated. In addition, we found that Porf-2 knockdown enhanced proliferation of the transplanted NSCs in retina, which may be due to the increased Wnt3a expression in retina after optic nerve injury. Thus, when optic nerve injury, the Wnt3a signaling is activated and Porf-2 plays an inhibitory role in such signaling transduction, which also explained the limited practical use of NSCs-based replacement therapy due to low proliferation rate. NSCs transplantation is reported to be a potential source of oncogenicity and tumor formation (Kaus et al., 2010). In the present study, we don’t found the tumorigenicity, indicating Porf-2 knockdown induces the increase of NSC proliferative capacity may be safe. But the tumorigenicity in longer term remains to be further observed.

Increased neurogenesis by the Wnt signaling pathway can be caused by increased proliferation of neuronally committed progenitors, or enhanced neuronal cell fate instruction (Lie et al., 2005). However, the Wnt/β-catenin pathway with Porf-2 knockdown or overexpression does not influence the neuronal and astrocytic differentiation of NSCs *in vitro* and *in vivo*, which is consistent with the previous reports (Ma and Nowak, 2011; Huang et al., 2016). One potential reason is that Rac1 activity changes. Rac1 regulates the self-renewal and differentiation of neural progenitor cells (Chen et al., 2009). Inhibition of Rac1 enhances NSC differentiation (Meng et al., 2015). Rac1 is dispensable in mediating proliferation and differentiation of SVZ progenitors (Leone et al., 2010). Rac1 deficiency causes an proliferation-induced SVZ-specific reduction along with increased cell cycle exit and premature differentiation. In telencephalic VZ progenitors, Rac1 deletion reduced the sizes of both cerebral cortex and the striatum. Further study has suggested that this abnormality is attributed to the accelerated cell cycle exit in early corticogenesis, causing a reduction of the neural progenitor pool (Chen et al., 2009). We infer that Rac1 activation increases during NSCs proliferation and decreases during differentiation.

Flash visual evoked potentials were analyzed in our study, as they quantify the degree of optic nerve injury and nerve conduction. The F-VEP latency was considered as an indictor in reflecting the function of nerve conduction and axon myelin sheath integrity, and the F-VEP amplitude in reflecting receptive function of the macula lutea and the number of intact synaptic contacts (Costa et al., 1985). Measuring F-VEP is objective and effective in evaluating optic nerve function. In our study, the data of F-VEP waveforms indicate the significant recovery of optic nerve function after NSCs transplantation. This result provided strong evidence that NSCs transplantation is a promising strategy in treating optic nerve injury. However, questions also follow. Whether a combination of NSCs transplantation and classic therapeutic strategies, such as steroid application and surgical decompression, is more effective requires further study. Post-transplantation, the peak latencies of the N1, P1, and N2 waves decreased, and the amplitude of N1-P1 and P1-N2 increased in the Porf-2 knockdown group compared with the control. The peak latencies of the N1, P1, and N2 waves increased, and the amplitude of N1-P1 and P1-N2 decreased in the Porf-2 overexpression group compared with the control. All of these findings suggest that Porf-2 downregulation contributes to the recovery of neurological functions, and conversely, Porf-2 overexpression decreases the number of differentiated neurons and impedes the recovery of neurological functions. The recovery of neurological functions was attribute for growth factor-derived NSCs and differentiated neurons which replace lost RGCs. In our study, the percentage of differentiation of NSCs into neurons failed to differ, but the increased number of NSCs by Porf-2 knockdown contributed to the increased number of neurons.

## Conclusion

Our results clearly suggest that Porf-2, a conserved RhoGAP, suppresses NSCs proliferation via the Rac1-Wnt/β-catenin pathway (summarized in **Figure 7D**). These findings provide greater insights into the effect of Porf-2 on NSCs proliferation and improve our understanding of the molecular and cellular mechanisms of Porf-2 control of NSCs proliferation, thus potentially contributing to the development of novel therapeutic strategies for improving visual function recovery. However, the clinical effects require further in-depth study.

## Author Contributions

X-TY and G-HH performed the experiments of morphology, histology, and biochemistry. H-JL and Z-LS have been involved in analysis and interpretation of data and in drafting and revising the manuscript critically. N-JX and D-FF designed experiments and revised the manuscript critically. All authors agree that all the questions related to the accuracy or integrity of the paper have been appropriately investigated and resolved, giving final approval of the version to be published.

## Conflict of Interest Statement

The authors declare that the research was conducted in the absence of any commercial or financial relationships that could be construed as a potential conflict of interest.

## Supplementary Material

FIGURE S1 Identification and differentiation of NSCs. **(a1–a4)** Representative phase-contrast photomicrographs of neurospheres derived from the hippocampus NSCs. **(b1–b4)** Quantification of the area of neurospheres and the number of neurospheres in different groups.
