# Supplementary figures and images for: Rac1 Guides Porf-2 to Wnt Pathway to Mediate Neural Stem Cell Proliferation
Source: Front Mol Neurosci. 2017 Jun 2;10:172. doi: 10.3389/fnmol.2017.00172 (PMC5454044; doi:10.3389/fnmol.2017.00172)

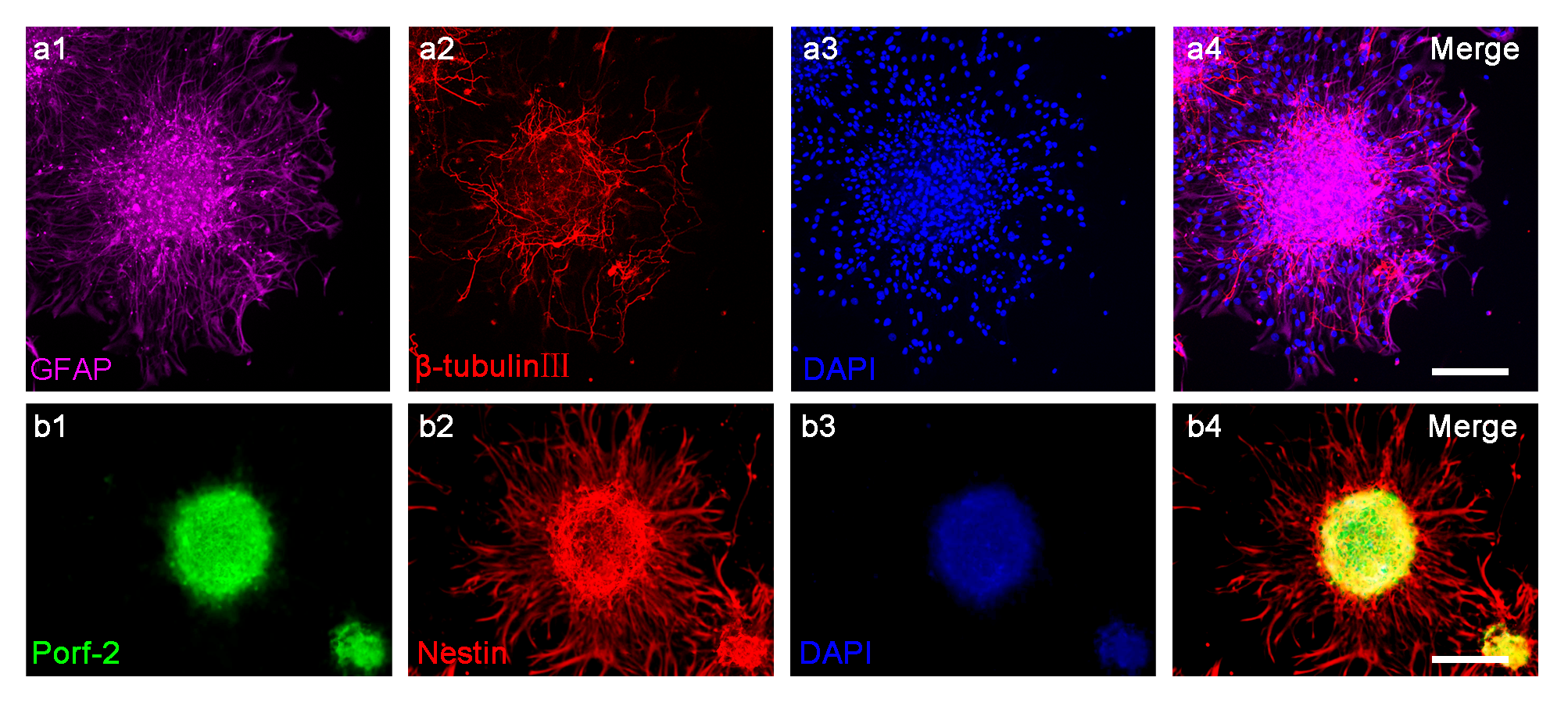

Supplement: FIGURE S1 — Identification and differentiation of NSCs. (a1–a4) Representative phase-contrast photomicrographs of neurospheres derived from the hippocampus NSCs. (b1–b4) Quantification of the area of neurospheres and the number of neurospheres in different groups. [file Image_1.TIF]
